# Lipoteichoic Acid from *Heyndrickxia coagulans* HOM5301 Modulates the Immune Response of RAW 264.7 Macrophages

**DOI:** 10.3390/nu16173014

**Published:** 2024-09-06

**Authors:** Shiqi Zhang, Pinglan Li, Xiao Zhang, Yan Ding, Tingting Wang, Suwon Lee, Ying Xu, Chongyoon Lim, Nan Shang

**Affiliations:** 1 College of Food Science and Nutritional Engineering, China Agricultural University, Beijing 100083, China; zsqkevin@163.com (S.Z.); lipinglan@cau.edu.cn (P.L.); 2 Food & Biotech R&D Center, Coree Beijing Co., Ltd., Beijing 101312, China; zhangxiao2@bjhanmi.com.cn (X.Z.); angeldingy@sina.com (Y.D.); wangtt@bjhanmi.com.cn (T.W.); leesw19@naver.com (S.L.); xy@bjhanmi.com.cn (Y.X.); chong_lim@naver.com (C.L.); 3 Key Laboratory of Precision Nutrition and Food Quality, Department of Nutrition and Healthy, China Agricultural University, Beijing 100083, China; 4 College of Engineering, China Agricultural University, Beijing 100083, China

**Keywords:** lipoteichoic acid, immunoadjuvant, *Heyndrickxia coagulans*, macrophages, boosting immunity, immunomodulatory mechanism

## Abstract

*Heyndrickxia coagulans* (formerly *Bacillus coagulans*) has been increasingly utilized as an immunomodulatory probiotics. Oral administration of *H. coagulans* HOM5301 significantly boosted both innate and adaptive immunity in mice, particularly by increasing the phagocytic capacity of monocytes/macrophages. Lipoteichoic acid (LTA), a major microbe-associated molecular pattern (MAMP) in Gram-positive bacteria, exhibits differential immunomodulatory effects due to its structural heterogeneity. We extracted, purified, and characterized LTA from *H. coagulans* HOM5301. The results showed that HOM5301 LTA consists of a glycerophosphate backbone. Its molecular weight is in the range of 10–16 kDa. HOM5301 LTA induced greater productions of nitric oxide, TNFα, and IL-6 in RAW 264.7 macrophages compared to *Staphylococcus aureus* LTA. Comparative transcriptome and proteome analyses identified the differentially expressed genes and proteins triggered by HOM5301 LTA. KEGG analyses revealed that HOM5301 LTA transcriptionally and translationally activated macrophages through two immune-related pathways: cytokine–cytokine receptor interaction and phagosome formation. Protein–protein interaction network analysis indicated that the pro-inflammatory response elicited by HOM5301 LTA was TLR2-dependent, possibly requiring the coreceptor CD14, and is mediated via the MAPK and NF-kappaB pathways. Our results demonstrate that LTA is an important MAMP of *H. coagulans* HOM5301 that boosts immune responses, suggesting that HOM5301 LTA may be a promising immunoadjuvant.

## 1. Introduction

*Heyndrickxia coagulans* (formerly classified as *Bacillus coagulans*), an endospore-forming and lactic acid-producing bacterium, is considered an ideal probiotic [1]. Several strains of *H. coagulans* have been demonstrated to improve host health by modulating immune homeostasis [2,3,4]. Spores, cell components, including peptidoglycans, teichoic acids (TAs), flagella, and metabolites, such as exopolysaccharides and proteins, serve as microbe-associated molecular patterns (MAMPs) that interact with pattern-recognition receptors (PPRs) expressed on immune cells, such as monocytes, dendritic cells, and natural killer (NK) cells [5,6]. The interaction between an MAMP and a PPR can lead to either pro-inflammatory or anti-inflammatory responses in the host by regulating the expression of chemokines, cytokines, and other signaling molecules [7].

Lipoteichoic acid (LTA), a major and unique component of the cell wall of Gram-positive bacteria, is a macro-amphiphile characterized by a hydrophilic backbone, typically composed of a 1,3-phosphodiester-linked polymer of glycerol–phosphate or ribitol–phosphate, variously substituted with D-alanine or hexose. The lipophilic region generally consists of a glycolipid, often a diglucosyl-diacylglycerol [8]. LTA is considered an analogue of lipopolysaccharide (LPS) of Gram-negative bacteria due to its similar pathophysiological properties [9]. However, accumulating evidence indicates that LTA interacts with Toll-like receptor 2 (TLR2) and initiates innate immune responses, in contrast to LPS, which is recognized by TLR4 [10,11]. Notably, the immunomodulatory properties of LTA are genus-, species-, and strain-specific. For instance, the LTAs of three *Apilactobacillus* strains showed significantly higher IgA-inducing activity compared to *Lactiplantibacillus plantarum* JCM1149T and *Lacticaseibacillus rhamnosus* GG in murine Peyer’s patch cells [12]. LTAs derived from *Lactiplantibacillus plantarum* A3, *Limosilactobacillus reuteri* DMSZ 8533, and *Lactobacillus acidophilus* CICC 6074 exhibited distinct anti-inflammatory activities in LPS-stimulated macrophages [13]. Kim et al. evaluated the effects of LTAs isolated from *Lactiplantibacillus plantarum* (LpLTA) and *Staphylococcus aureus* (SaLTA) on chemokine (C–C motif) ligand 2 (CCL2) production in THP-1 cells. LpLTA significantly inhibited SaLTA-mediated CCL2 production via the TLR2 pathway [10]. Variations in the molecular structures of LTAs, such as the type and degree of substitution and the length of the backbone, result in diverse immunostimulatory activities [14].

In a previous study, we demonstrated that daily administration of *H. coagulans* HOM5301 for one month significantly enhanced both non-specific and specific immunity in mice, particularly the phagocytic capacity of monocytes/macrophages [15]. We hypothesized that LTA is a key molecule through which *H. coagulans* HOM5301 enhances immunity. This study investigated the immunomodulatory properties and potential molecular mechanisms of action of LTA derived from *H. coagulans* HOM5301 in macrophages. To this end, we examined the effects of LTA on the activation and pro-inflammatory functions of RAW 264.7 cells. Comparative transcriptome and proteome analyses were conducted to identify differentially expressed genes (DEGs) and proteins induced by LTA. Several immune-related pathways enriched by LTA were identified. We hope that our findings provide valuable insights for future studies on the immunomodulatory properties of *H. coagulans* and its LTA.

## 2. Materials and Methods

### 2.1. Materials and Chemicals

*H. coagulans* HOM5301 were deposited in the China General Microbiological Culture Collection Center (CGMCC; No. 20383) and cultured with De Man, Rogosa, and Sharpe media (MRS; Oxiod, Hants, UK) in a shaker at 200 rpm. The RAW 264.7 macrophages were purchased from the Cell Bank of the Chinese Academy of Sciences, and cultured in Dulbecco’s Modified Eagle’s Medium (DMEM; Thermo Fisher Scientific, Waltham, MA, USA) with 10% fetal bovine serum (FBS) and 1% streptomycin–penicillin solution. LPS (Cat. No. L2880) derived from *Escherichia coli* 055:B5 and SaLTA (Cat. No. L2515) were purchased from Sigma-Aldrich (Merck, Germany). MTS reagent, 3-(4,5-dimethylthiazol-2-yl)-5-(3-carboxymethoxyphenyl)-2-(4-sulfophenyl)-2H-tetrazolium, was purchased from Promega (Madison, WI, USA). The detection kit for nitric oxide (NO) was obtained from Nanjing Jiancheng Bioengineering Institute (Jiangsu, China). The Enzyme-linked immunosorbent assay (ELISA) kits for tumor necrosis factor α (TNFα), interleukin-6 (IL-6), and interleukin-10 (IL-10) were purchased from RayBiotech (Norcross, GA, USA).

### 2.2. Preparation of LTA

Extraction and purification of LTA were performed as described by Balaguer et al. [16], with some modifications. Overnight, *H. coagulans* HOM5301 cell cultures were centrifuged (6000× *g*, 20 min) and washed three times with 0.1 M Tris-HCl buffer. The bacterial pellet was resuspended in 0.1 M ammonium acetate buffer (pH 4.7) and mixed with an equal volume of n-butanol. The solution was incubated in a shaker at 37 °C and 200 rpm for 1 h, and then centrifuged at 12,000× *g* for 15 min. The aqueous phase containing LTA was retrieved and loaded onto a hydrophobic interaction chromatography column (octyl agarose gel CL-4B) that was pre-equilibrated with 0.1 M ammonium acetate buffer (pH 4.7). Subsequently, the column was eluted with 15% and 35% n-propanol solutions. Fractions containing LTA were identified using a phosphate-content-determination assay, using ammonium molybdate [17]. The phosphate-positive fractions eluted with 35% n-propanol were dialyzed at 4 °C for 72 h and freeze-dried for further use.

### 2.3. Characterization of LTA

The homogeneity of LTA was analyzed using high-performance liquid chromatography (HPLC) combined with a refractive index detector (RID) and a size-exclusion chromatography (SEC) column (BioCore SEC300, NanoChrom, Suzhou, China), according to the method described by Liu et al. [18]. The relative molecular weight was determined using matrix-assisted laser desorption ionization time-of-flight (MALDI-TOF) mass spectrometry (Bruker Ultraflextreme, Bremen, Germany). Two mass ranges (0–10,000 *m*/*z* and 10,000–100,000 *m*/*z*) were detected [16]. Fourier-transform infrared (FT-IR) spectroscopy and nuclear magnetic resonance (NMR) spectrometry were employed to identify the structure of LTA, as previously described [19,20]. Briefly, the transmission FT-IR spectra of LTA were collected 4000–400 cm^−1^ using a FT-IR spectrometer (Nicolet 6700, Thermo Fisher Scientific, Waltham, MA, USA), using the KBr pressed-disk method. ^1^H NMR spectra in D_2_O solvent were recorded using a Bruker AV-600 NMR spectrometer (Rheinstetten, Germany). SaLTA was used as a control.

### 2.4. Macrophage Proliferation Assay

Cell viability was determined using the MTS assay [21]. RAW 264.7 cells were seeded at 5.0 × 10^5^ cells/mL in 96-well plates with DMEM, supplemented with 10% FBS (*v*/*v*) and 1% penicillin–streptomycin solution, and incubated at 37 °C with 5% CO_2_ for 4 h. Then the spent culture medium was replaced with an increasing concentration of LTA (6.25, 12.5, 25, 50, 100, and 200 μg/mL) dissolved in fresh DMEM and incubated for another 24 h. After treatment, 20 μL of MTS reagent was added directly to the wells and incubated for another 4 h. The absorbance (optical density, OD) was measured at 490 nm using a microplate reader. LPS (1 μg/mL) and SaLTA (100 μg/mL) dissolved in complete DMEM were set as positive controls [9]. DPBS was set as a negative control (NC), and the proliferative activity was defined as 100%. Proliferative activity (%) = OD (sample)/OD (negative control) × 100%.

### 2.5. Macrophage Stimulation Assay

RAW 264.7 cells were seeded at 5.0 × 10^5^ cells/mL in 24-well plates with DMEM, supplemented with 10% FBS (*v*/*v*) and 1% penicillin–streptomycin solution, and incubated at 37 °C with 5% CO_2_ for 4 h. Then, the spent culture medium was replaced with an increasing concentration of LTA (25, 50, and 100 μg/mL) dissolved in complete DMEM, and incubated for another 24 h. LPS (1 μg/mL) and SaLTA (100 μg/mL) dissolved in complete DMEM were set as positive controls [9]. The culture supernatants were collected by centrifugation at 1500× *g* for 8 min. The NO content and three cytokines, TNFα, IL-6, and IL-10, were measured using detection kits, according to the manufacturer’s protocols.

### 2.6. Transcriptome and Proteome Analyses

RAW 264.7 cells were seeded at 5.0 × 10^5^ cells/mL in 6-well plates with complete DMEM, and incubated at 37 °C in a 5% CO_2_ environment for 4 h. The spent culture medium was then replaced with fresh complete DMEM containing 100 μg/mL of LTA, and incubated for another 24 h. Complete DMEM was used as a negative control. The cells were harvested and rinsed three times with DPBS. Total RNA was extracted using TRIzol reagent (TIANGEN, Beijing, China). The samples were sent to Majorbio Bio-Pharm Biotechnology Co., Ltd. (Shanghai, China) for RNA-seq and four-dimensional data-independent acquisition (4D-DIA) proteomic sequencing.

#### 2.6.1. RNA-seq Data Processing and Analysis

RNA-seq experiments were performed using the Illumina NovaSeq X Plus platform (PE150) and NovaSeq Reagent Kit (San Diego, CA, USA). Quality-controlled reads were obtained by removing reads containing ploy-N and adapter, and low-quality reads (quality score < 20), using the fastp software (version 0.19.5) [22]. Then, processed reads were aligned to the *Mus musculus* reference genome in orientation mode using HISAT2 software [23]. The transcriptome of each sample was reassembled using StringTie version 2.1.2 [24]. Differential expression analysis was performed using DESeq2 software (version 1.24.0) [25]. The *p*-value was adjusted by the false discovery rate (FDR) < 0.05. A corrected *p*-value of 0.05 and a |log2-fold change| of 1 were selected as the default thresholds for the significant DEGs. The Kyoto Encyclopedia of Genes and Genomes (KEGG) database was employed to identify pathways that were significantly enriched by HOM5301 LTA treatment. Pathways were considered significantly enriched in DEGs when the *p*-adjust was less than 0.05.

#### 2.6.2. Proteomic Data Processing and Analysis

The proteomic data obtained by liquid chromatography with tandem mass spectrometry (LC-MS-MS) were processed and analyzed using the online Majorbio Cloud Platform [26]. The MS/MS spectra were matched to the UniProt database. The abundance of each protein in each sample was normalized to the average abundance of the protein in all samples to obtain the relative protein abundance ratio for further analysis. Differential expression analysis was performed using Student’s *t*-test in R software (version 4.2.1) [27]. An adjusted *p* < 0.05 and a |log2FC| of ≥1 were set as the thresholds for the significant differentially expressed proteins (DEPs). The DEPs identified between the LTA and negative control groups were annotated using the KEGG database. Pathways were defined as significantly enriched in DEPs when the *p*-adjust was less than 0.05.

#### 2.6.3. Protein–Protein Interaction Networks Analysis

The DEPs related to TLR and cytokine–cytokine interaction pathways were selected to construct a protein–protein interaction (PPI) network using the STRING database (STRING: functional protein association networks, http://string-db.org/, accessed on 10 April 2024.). The relative expression levels of several key node genes and proteins in the PPI network were randomly selected for statistical analysis, respectively.

### 2.7. Statistical Analysis

All values are presented as mean ± standard deviation. Statistical significance was determined using GraphPad Prism version 8. Significances between multiple groups were analyzed using one-way analysis of variance followed by the Tukey multiple comparison test. The significance between two groups was assessed using Student’s *t*-test. Statistical significance was determined at *p* < 0.05.

## 3. Results

### 3.1. Characterization of LTA

**Figure 1:**
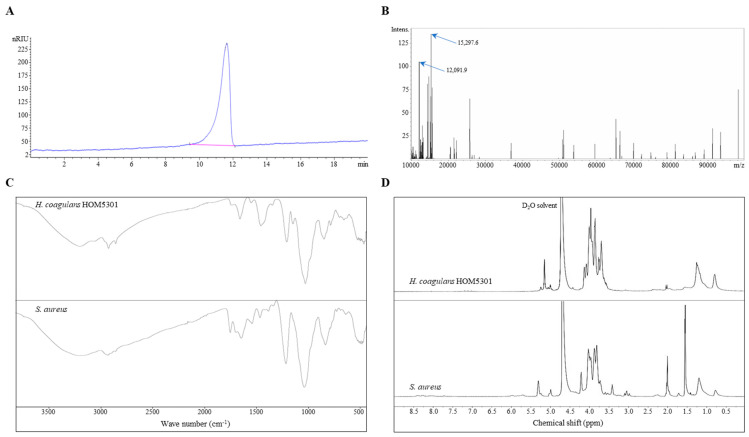
Properties of LTA isolated from *H. coagulans* HOM5301. (**A**) HPLC-SEC-RID analysis of the purified LTA. (**B**) MALDI-TOF mass spectrum of purified LTA (10,000–100,000 *m*/*z*). (**C**) FT-IR spectra of purified LTA and SaLTA. (**D**) ^1^H-NMR spectra of purified LTA and SaLTA in D_2_O solvent (600 MHz).

LTA from *H. coagulans* HOM5301 was purified using octyl agarose gel CL-4B, as described in the Methods section, and analyzed using HPLC-SEC-RID. A single concentrated peak was observed using HPLC, with LTA eluting from the column after approximately 11 min (Figure 1A).

The results of MALDI-TOF mass spectrometry showed that there was no significant response signal between 0 and 10,000 *m*/*z*, aside from the solvent peaks. However, characteristic peaks were primarily observed between 10,000–16,000 *m*/*z* in the 10,000–100,000 *m*/*z* range (Figure 1B).

A comparison between LTA and SaLTA is shown in Figure 1C. Several characteristic peaks of the two samples appeared at the same positions. The peak at 2920 cm^−1^ was attributed to the -OH group. The stretching vibration of the amide group was recorded at 1654 cm^−1^. The band at 1202 cm^−1^ corresponded to the vibration of the P=O bond. The bands at 1023 cm^−1^ indicated vibrations from amino acids, sugars, and other residues. 

Figure 1D shows the ^1^H-NMR spectra of LTA and SaLTA. The major differences between the two proton NMR spectra were in the chemical shifts (δ)_H_ = 1.5, 2.0, and 3.5–4.0. The singlet at δ_H_ = 5.1 was attributed to the -OH group. The -CH-group proton peak appeared at δ_H_ = 4.2. The ^1^H of the sugar residue showed a broad signal near 4.0 ppm. Peaks at 2.0–2.1 and 1.5 ppm were derived from the resonances of -CH_2_ and -CH_3_, respectively.

### 3.2. Influence on Macrophage Proliferation

**Figure 2:**
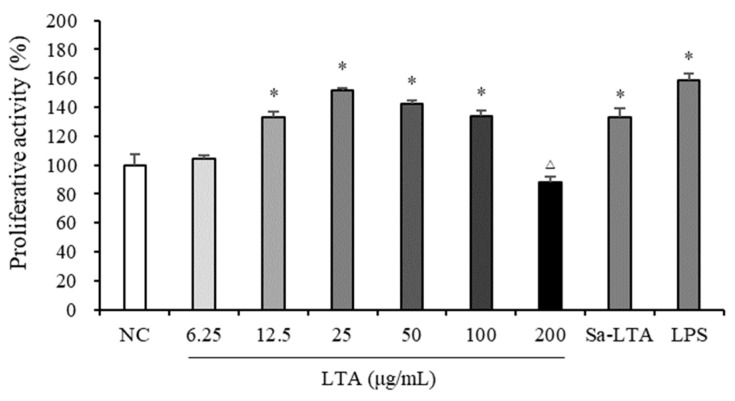
Proliferative effects of LTA on macrophages. * indicates significantly higher than the negative control (NC) (*p* < 0.05); △ indicates significantly lower than the NC (*p* < 0.05).

The MTS assay was performed to measure the proliferative effect and cytotoxicity of LTA in macrophages, by determining cell viability (Figure 2). Compared to the negative control (NC), LTA significantly promoted macrophage proliferation at concentrations between 12.5 and 100 μg/mL. However, LTA showed a cytotoxic effect on macrophages at a concentration of 200 μg/mL. Therefore, three concentrations (25, 50, and 100 μg/mL) were selected to assess the immunostimulatory activity of LTA on macrophages.

### 3.3. Effects on Macrophage Stimulation

**Figure 3:**
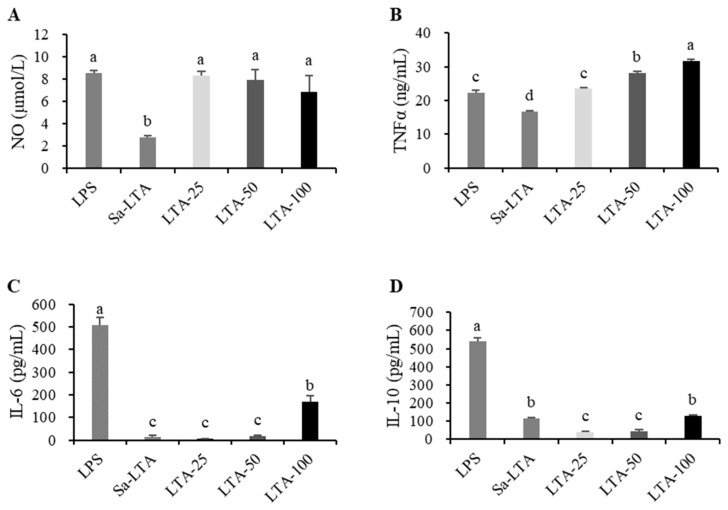
Effects of LTA on the production of NO (**A**), TNFα (**B**), IL-6 (**C**), and IL-10 (**D**) in RAW 264.7 cells. The different letter labels on error bars represent statistically significant differences among the tested samples (*p* < 0.05).

To evaluate the effect of LTA on macrophage stimulation, four indicators were measured: nitric oxide (NO), TNFα, IL-6, and IL-10 (Figure 3). The three tested concentrations of LTA (25, 50, and 100 μg/mL) showed similar abilities to promote NO production, comparable to 1 μg/mL LPS (*p* > 0.05), and significantly higher than 100 μg/mL SaLTA (*p* < 0.05). LTA increased TNFα expression in a dose-dependent manner, with both the 50 and 100 μg/mL concentrations inducing higher TNFα production than LPS (*p* < 0.05). Additionally, LTA induced greater secretion of TNFα than SaLTA (*p* < 0.05). While LTA increased the release of IL-6, it did so to a lesser extent than LPS. At the same concentration (100 μg/mL), LTA induced higher IL-6 production than SaLTA. In contrast, LTA did not significantly enhance the expression of the anti-inflammatory cytokine IL-10 compared to LPS or SaLTA. A concentration of 100 μg/mL was selected for further analyses.

### 3.4. Overall Analysis of Gene and Protein Expression

**Figure 4:**
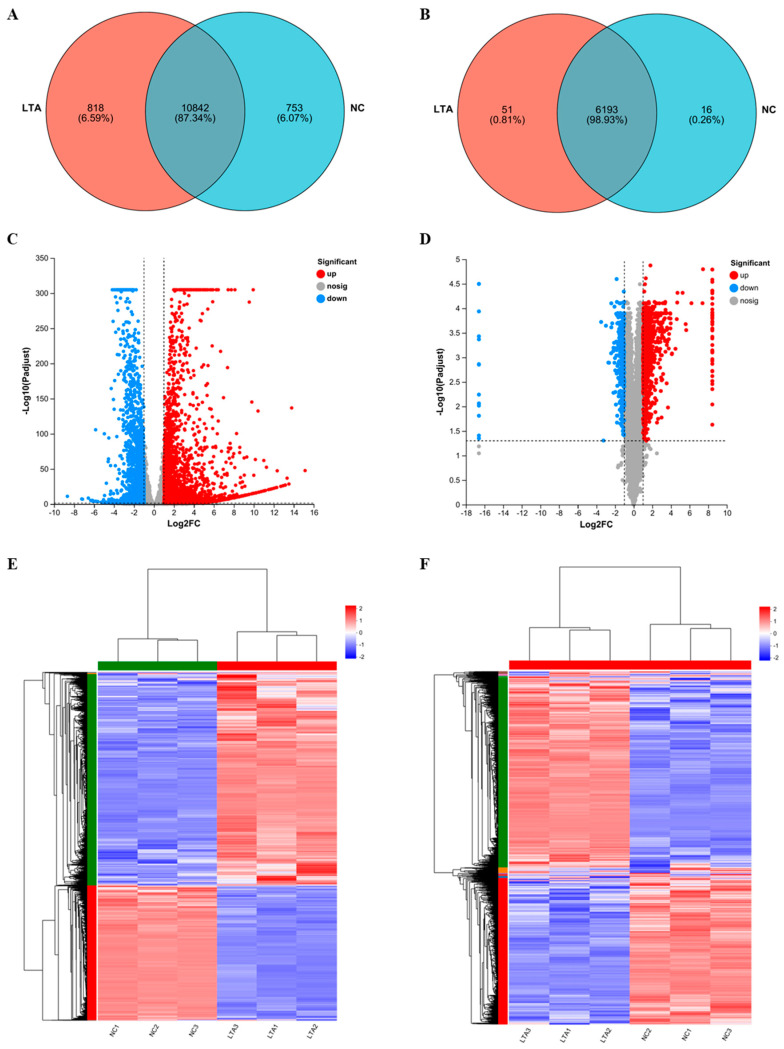
Differential expression patterns of mRNAs and proteins between the negative control and LTA-treated RAW 264.7 macrophages. (**A**) Venn diagram of genes co-expressed between the NC and LTA-treated groups. (**B**) Venn diagram of proteins co-expressed between the NC and LTA-treated groups. (**C**) Volcano plot of all sequenced genes from the NC and LTA-treated groups. (**D**) Volcano plot of all sequenced proteins from the NC and LTA-treated groups. (**E**) Heat map of differentially expressed genes (DEGs) between the NC and LTA-treated groups. (**F**) Heat map of differentially expressed proteins (DEPs) between the NC and LTA-treated groups.

RNA-seq and 4D-DIA proteomic sequencing were employed to identify changes in gene and protein expression induced by LTA treatment in RAW 264.6 cells, and to further elucidate the molecular mechanisms underlying LTA’s immunomodulatory activities. At the gene-transcription level, 10,842 genes were co-transcribed in both the control and LTA-treated groups, while 818 genes were exclusively transcribed in the LTA-treated group (Figure 4A). The overall distribution of DEGs is displayed as a volcano plot in Figure 4C, revealing that LTA treatment up-regulated 2924 genes, and down-regulated 1826 genes.

At the protein expression level, 6193 proteins were co-expressed in both groups, while 51 proteins were uniquely expressed in the LTA-treated group (Figure 4B). Moreover, 1111 DEPs were identified, as illustrated by the volcano plot in Figure 4D, with 843 up-regulated and 268 down-regulated by LTA. Heat maps were generated to display clusters of DEGs (Figure 4E) and DEPs (Figure 4F) between the NC and LTA-treated groups, demonstrating consistent clustering among those three biological replicates for each treatment.

### 3.5. Functional Enrichment Analysis of DEGs and DEPs

**Figure 5:**
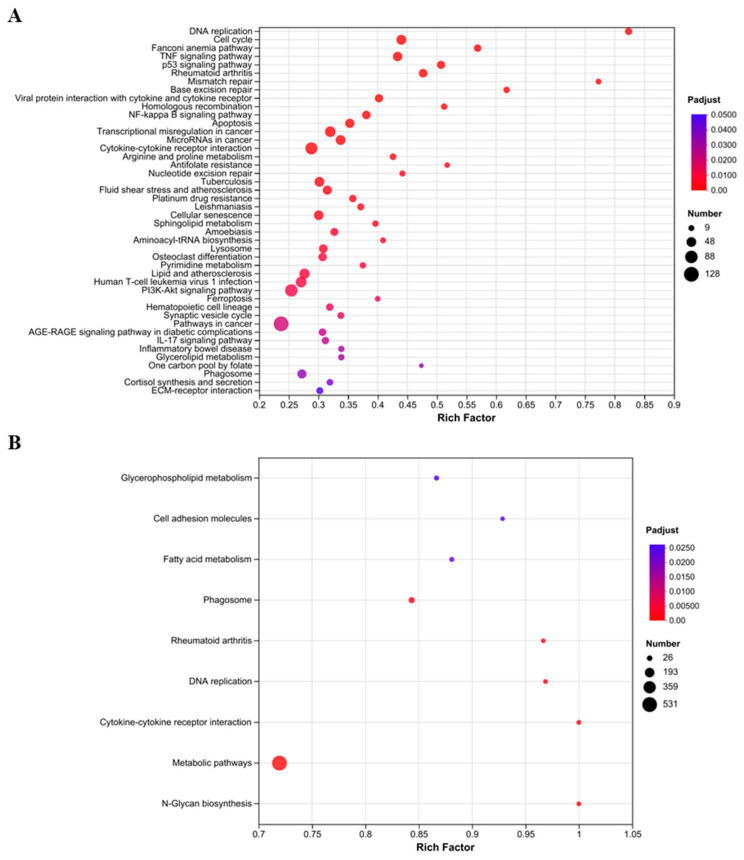
Functional enrichment analysis of DEGs and DEPs based on KEGG pathway category. (**A**) Scatterplot of significantly enriched KEGG pathways in DEGs (*p*-adjust < 0.05). (**B**) Scatterplot of significantly enriched KEGG pathways in DEPs (*p*-adjust < 0.05). The color and size of the dots represent the *p*-adjust values and DEP numbers, respectively.

To investigate the implications of DEGs and DEPs, and to elucidate the potential regulatory mechanisms in LTA-exposed RAW 264.7 macrophages, KEGG functional enrichment analysis was performed. The LTA-treated macrophages exhibited 44 significantly enriched KEGG pathways based on DEGs (Figure 5A), while the NC group exhibited only nine significantly enriched KEGG pathways based on DEPs (Figure 5B). At the gene expression level, immune-related pathways including the nuclear factor-kappa B (NF-κB) signaling pathway, cytokine–cytokine receptor interaction, viral protein interaction with cytokine and cytokine receptors, the TNF signaling pathway, a phagosome, the IL-17 signaling pathway, and the phosphatidylinositol 3-kinase (PI3K)/protein kinase B (PKB/AKT) signaling pathway were significantly enriched (*p*-adjust < 0.05). At the protein-translation level, only two immune-related pathways, cytokine–cytokine receptor interaction and phagosomes, were significantly enriched (*p*-adjust < 0.05), consistent with the results observed at the gene expression level.

### 3.6. PPI Network Analysis

**Figure 6:**
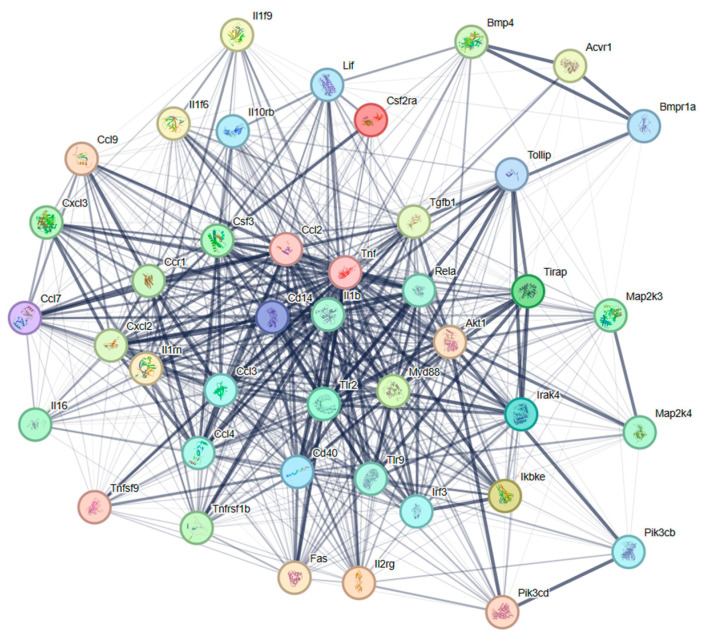
PPI network of DEPs associated with TLR signaling and cytokine–cytokine interaction pathways. The network nodes represent the DEPs, and the lines indicate associations between the linked DEPs.

**Figure 7:**
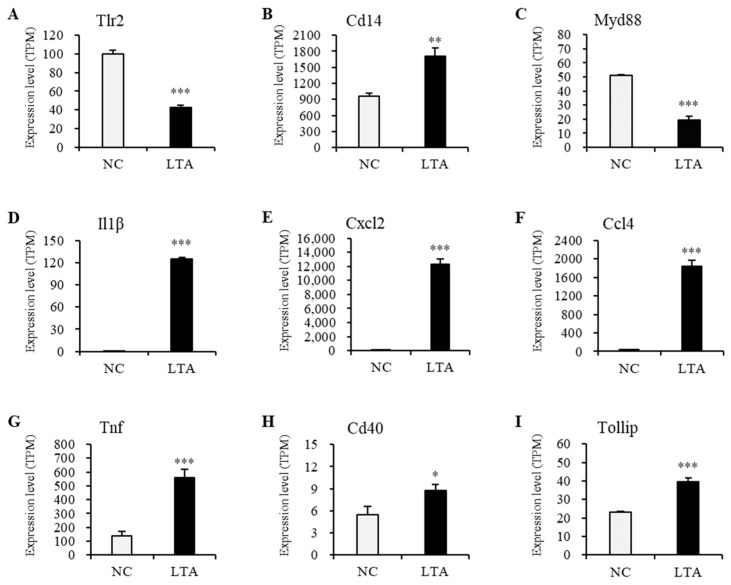
Relative expression levels of the nine selected genes in the PPI network with significant differences between the negative control and LTA-treated RAW 264.7 macrophages. (**A**) Tlr2. (**B**) Cd14. (**C**) Myd88. (**D**) Il1β. (**E**) Cxcl2. (**F**) Ccl4. (**G**) Tnf. (**H**) Cd40. (**I**) Tollip. Quantification results were normalized using transcripts per million. * *p* < 0.05, ** *p* < 0.01, and *** *p* < 0.001 vs. NC.

**Figure 8:**
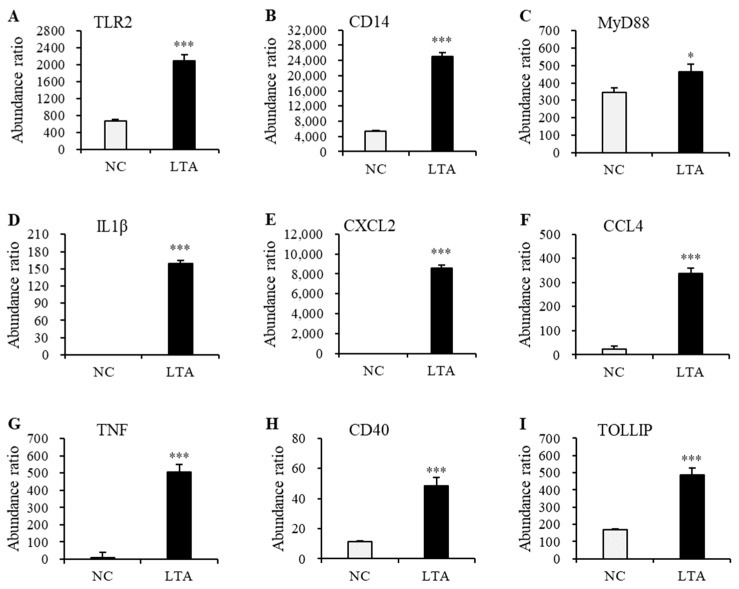
The expression levels of the nine selected proteins in the PPI network with significant differences between the negative control and LTA-treated RAW 264.7 cells. (**A**) TLR2. (**B**) CD14. (**C**) MyD88. (**D**) IL1β. (**E**) CXCL2. (**F**) CCL4. (**G**) TNF. (**H**) CD40. (**I**) TOLLIP. * *p* < 0.05 and *** *p* < 0.001 vs. NC.

To explore the potential regulatory mechanisms in LTA-exposed RAW 264.7 macrophages, DEPs related to TLR signaling and cytokine–cytokine interaction pathways were mapped onto the STRING database for the PPI network. As shown in Figure 6, the PPI network consisted of 42 nodes. Nine widely reported nodes were randomly selected for comparative analyses of gene expression (Figure 7) and protein expression (Figure 8). 

The gene expression levels of *Tlr2* and *Myd88* in macrophages treated with LTA for 24 h were significantly lower than those in the NC group. However, protein expression levels of TLR2 and MyD88 in LTA-treated macrophages were significantly higher than those in the NC group. Additionally, the gene expression levels of *Cd14*, *Il1β*, *Cxcl2*, *Ccl4*, *Tnf*, *Cd40*, and *Tollip*, along with their corresponding protein expression levels, were significantly increased in the LTA treatment group (*p* < 0.05).

## 4. Discussion

Lipoteichoic acid (LTA), a component of the cell wall, is considered a critical molecule for the immunomodulatory effects of probiotics [28]. Variations in the molecular structure of LTAs from different probiotic strains lead to diverse immunostimulatory activities. For instance, Claes et al. reported that LTA is an important MAMP in *Lacticaseibacillus rhamnosus* GG (LGG) with pro-inflammatory activities [17]. Conversely, *L. plantarum* NCIMB8826 LTA has been reported to exhibit anti-inflammatory properties [29]. To select the optimal strain for specific applications, it is essential to understand the detailed molecular mechanisms of action. 

In a previous study, we reported that daily administration of *H. coagulans* HOM5301 for one month significantly boosted both non-specific and specific immunity in mice, especially the phagocytic capacity of monocytes/macrophages [15]. In this study, multi-spectrometric analyses preliminarily indicated that HOM5301 LTA consists of a glycerophosphate backbone with amide, hexose, and amino acid substituents, and other groups, with a molecular weight range of 10–16 kDa. HOM5301 LTA is structurally distinct from SaLTA [30]. Furthermore, HOM5301 LTA efficiently activated macrophages, promoting the expression of NO, TNFα, and IL-6 to a greater extent than SaLTA, a potent inducer of inflammation [31,32]. This suggests that HOM5301 LTA has great potential as an immune-boosting agent. Given the complexity of LTA’s structure, further research is needed to elucidate its chemical structure and structure-activity relationship.

To elucidate the mechanism by which HOM5301 LTA activates macrophages, we employed RNA sequencing and proteomic approaches. The up-regulation of key pro-inflammatory genes detected by RNA-seq was corroborated by proteomic analysis, indicating a strong activation effect of HOM5301 LTA on macrophages. After 24 h of LTA treatment, we observed greater differential expression at the gene level than at the protein level. Interestingly, the expression levels of *Tlr2* and *Myd88* in LTA-treated macrophages were significantly lower at the gene level, compared to the NC group, while the corresponding protein levels were significantly higher. This discrepancy may be due to the time lag between gene transcription and protein translation. Future studies should analyze gene expression during the early stages of LTA treatment.

Phagocytosis, primarily performed by neutrophils, monocytes, and macrophages, is a fundamental defense mechanism in organisms and is a component of non-specific immunity [33]. The synergistic interaction between the mononuclear-phagocyte and lymphocyte systems forms the basis of specific immune responses (humoral and cellular immunity) [34]. A key step in phagocytosis is the formation of phagosomes, which are proposed as organelles that link innate and adaptive immunity [35]. Cytokine–cytokine receptor interactions regulate the immune system, including cell growth, differentiation, and immune responses. For example, different types of cytokines, such as ILs, interferons, TNFs, and chemokines, can stimulate the proliferation and differentiation of immune cells by binding to specific receptors, thereby enhancing or inhibiting immune responses [36]. Through KEGG analysis, we confirmed that HOM5301 LTA transcriptionally and translationally activated macrophages via two immune-related pathways: cytokine–cytokine receptor interactions and phagosomes.

**Figure 9:**
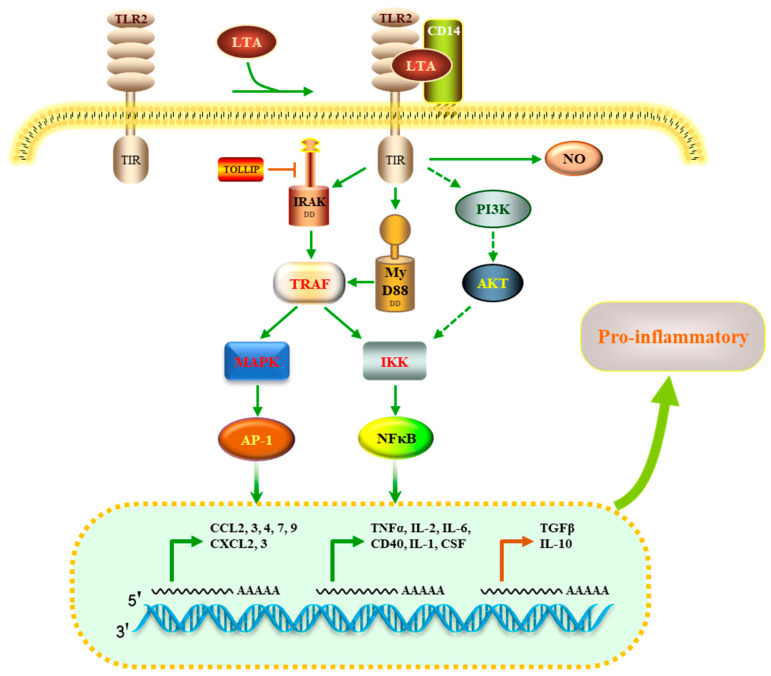
Proposed mechanisms for the immunomodulatory activity of HOM5301 LTA on macrophages.

Several studies have reported that LTA interacts with TLR2 to initiate innate immune responses [17,29]. To determine the molecular mechanism by which HOM5301 LTA acts on macrophages, we constructed a PPI network using DEPs involved in TLR signaling and cytokine–cytokine interaction pathways. The proposed mechanism of action is illustrated in Figure 9. HOM5301 LTA stimulates TLR2, recruiting MyD88 and IRAK, which subsequently trigger downstream cascades leading to the activation of mitogen-activated protein kinases (MAPK) and NF-κB. This cascade ultimately induces the expression of pro-inflammatory cytokines, such as TNF, IL6, IL1β, CD40, CSF, and chemokines, including CXCL2, CXCL3, CCL2, and CCL4. The pro-inflammatory properties of HOM5301 LTA are TLR2-dependent, but may require the coreceptor CD14. This finding is similar to that of Nilsen et al., who found that the pro-inflammatory property of SaLTA was TLR2 dependent and required the coreceptors CD14 and CD36 [37]. Additionally, we found that several negative regulators of pro-inflammation, such as the Toll-interacting protein, transforming growth factor beta, and IL-10, were up-regulated, which helps prevent exaggerated inflammatory responses caused by HOM5301 LTA. This is consistent with the immunoregulatory characteristics of LTA from LGG in bone marrow-derived dendritic cells [9]. Further studies on HOM5301 LTA in gut-associated lymphoid tissue and in mice are required to better understand the mechanism of action of *H. coagulans* in enhancing immunity.

## 5. Conclusions

In this study, we extracted, purified, and characterized LTA from *H. coagulans* HOM5301. The results showed that HOM5301 LTA consists of a glycerophosphate backbone. Its molecular weight is in the range of 10–16 kDa. Comparative transcriptome and proteome analyses showed that LTA is an important MAMP of *H. coagulans* HOM5301, which activates macrophages via extracellular interactions with TLR2. The MAPK and NF-κB pathways are crucial to the LTA-induced immunomodulatory effect on macrophages, which partly explains the mechanism of action of *H. coagulans* HOM5301 in boosting immunity. The application of LTA derived from *H. coagulans* as an immunoadjuvant shows potential; however, more in-depth and extensive research is required.

## Data Availability

The raw data supporting the conclusions of this article will be made available by the authors upon request.
